# Substituting mouse transcription factor Pou4f2 with a sea urchin orthologue restores retinal ganglion cell development

**DOI:** 10.1098/rspb.2015.2978

**Published:** 2016-03-16

**Authors:** Chai-An Mao, Cavit Agca, Julie A. Mocko-Strand, Jing Wang, Esther Ullrich-Lüter, Ping Pan, Steven W. Wang, Maria Ina Arnone, Laura J. Frishman, William H. Klein

**Affiliations:** 1 Department of Systems Biology, Unit 0950, The University of Texas MD Anderson Cancer Center, Houston, TX 77030, USA; 2 College of Optometry, University of Houston, Houston, TX 77204, USA; 3 Museum für Naturkunde, Berlin 10115, Germany; 4 Biology and Evolution of Marine Organisms, Stazione Zoologica Anton Dohrn, Naples 80121, Italy

**Keywords:** retinal ganglion cells, retinal development, transcription factor evolution, Pou4f2/Brn3b, echinoderm photoreceptors

## Abstract

Pou domain transcription factor Pou4f2 is essential for the development of retinal ganglion cells (RGCs) in the vertebrate retina. A distant orthologue of Pou4f2 exists in the genome of the sea urchin (class Echinoidea) *Strongylocentrotus purpuratus* (*SpPou4f1/2*), yet the photosensory structure of sea urchins is strikingly different from that of the mammalian retina. Sea urchins have no obvious eyes, but have photoreceptors clustered around their tube feet disc. The mechanisms that are associated with the development and function of photoreception in sea urchins are largely unexplored. As an initial approach to better understand the sea urchin photosensory structure and relate it to the mammalian retina, we asked whether *SpPou4f1/2* could support RGC development in the absence of *Pou4f2*. To answer this question, we replaced genomic *Pou4f2* with an *SpPou4f1/2* cDNA. In *Pou4f2*-null mice, retinas expressing *SpPou4f1/2* were outwardly identical to those of wild-type mice. *SpPou4f1/2* retinas exhibited dark-adapted electroretinogram scotopic threshold responses, indicating functionally active RGCs. During retinal development, *SpPou4f1/2* activated RGC-specific genes and in *S. purpuratus*, *SpPou4f2* was expressed in photoreceptor cells of tube feet in a pattern distinct from Opsin4 and Pax6. Our results suggest that *SpPou4f1/2* and *Pou4f2* share conserved components of a gene network for photosensory development and they maintain their conserved intrinsic functions despite vast morphological differences in mouse and sea urchin photosensory structures.

## 1. Introduction

The deep conservation of regulatory genes for eye development has amply demonstrated an underlying framework for eye diversification [1,2]. However, the developmental and evolutionary mechanisms that led to this remarkable diversity remain vague. Although changes in gene regulatory networks are probably to be the drivers of eye diversification, very little is known about the level at which conserved gene networks might have diverged to produce different structures and functions [2]. A potentially informative but largely unexplored phylum for investigating eye development and evolution is echinoderms. Echinoderms are basal deuterostomes that develop in ways similar to chordates but are distinctly different in their adult body plan. Unlike all other deuterostomes, echinoderms lack an obvious anterior–posterior axis. Instead, they exhibit highly derived body plans that are organized along a radial axis [3]. Notably, echinoderms lack any structures that even remotely resemble a vertebrate eye. Nevertheless, many behavioural patterns in adult sea urchins are attributable to highly developed photoreception [4].

We recently addressed the question of whether adult echinoderms have distinct photosensitive neurons that are separate from the diffuse surface-wide neural network already known to exist [5–7]. Using probes that were *Strongylocentrotus purpuratus* orthologues of mouse genes expressed in the retina [8], Agca *et al*. [9] and Ullrich-Lüter *et al*. [10] showed that small groups of photoreceptor neurons were clustered around the periphery of the tube feet disc. This conclusion was based on the fact that many genes expressed in the mouse retina had homologues that were expressed in the tube feet neurons. The genes included retinal-expressed transcription factors [8–12] and an orthologue of opsin, *Opsin4*, which had signature features of a light-transducing opsin [8–11,13]. Many of the transcription factors have important roles in the developing mouse retina. Of particular note, the *S. purpuratus* orthologue of *Pou4f2*, *SpPou4f1/2*, has a 91% and 96.6% sequence match with mouse *Pou4f2* in its Pou-specific and Pou-homeodomains, respectively. However, it is highly divergent outside of these domains (electronic supplementary material, figure S1*a*). Phylogenetic analysis of *Pou*-class genes showed that *SpPou4f1/2* clustered with the *Pou4f2* family over these domains [8]. We have shown that *SpPou4f1/2* is expressed in the tube feet [9].

Although the results of Agca *et al*. [9] and Ullrich-Lüter *et al*. [10] are correlative, they suggest the presence of functional photoreceptor neurons in the tube feet. However, the function of *SpPou4f1/2* in the tube feet is unknown. Given the technical difficulties in directly determining the function of *SpPou4f1/2* in the adult sea urchin tube feet, we chose a more feasible, albeit less direct, approach using a mouse knock-in (KI) strategy. We asked whether *SpPou4f1/2* could function in the context of the developing mouse retina. *Pou4f2* is essential for retinal ganglion cell (RGC) differentiation and survival [14–16]. In the developing retina, *Pou4f2* expression is restricted to newly differentiated RGCs, and its expression is maintained throughout adult life. If *SpPou4f1/2* functionally replaced *Pou4f2*, it would suggest that SpPou4f1/2 binds to and activates a similar set of genes in tube feet neurons, despite more than 540 Myr of divergence from the common ancestor of sea urchins and mice.

As demonstrated further in the article, our experiments in both mice and sea urchins support this hypothesis and indicate that conservation of Pou-specific and Pou-homeodomains in *SpPou4f1/2* is, to high degree, sufficient to support RGC development.

## 2. Material and methods

### (a) Pou4 class protein sequence analysis

We performed protein sequence analysis with Megalign software (DNASTAR, Madison, WI, USA) using the ClustalW multiple sequence alignment method using four protein sequences: mouse Pou4f1, Pou4f2, Pou4f3 and *S. purpuratus* SpPou4f1/2. Phylogeny tree was constructed with MEGA5 software [17] using neighbour-joining method with 1000 bootstrap repeats. Amino acid sequences from multiple POU families were used to construct a phylogeny tree [18]. Sequences from *Ciona intestinalis*, *Danio rerio*, *Mus Musculus*, *Strogylocentrotus purpuratus* and *Xenopus tropicalis* were used for comparison.

### (b) Generation of *Pou4f2^SpPou4f1/2^* knock-in construct

**Figure 1.**
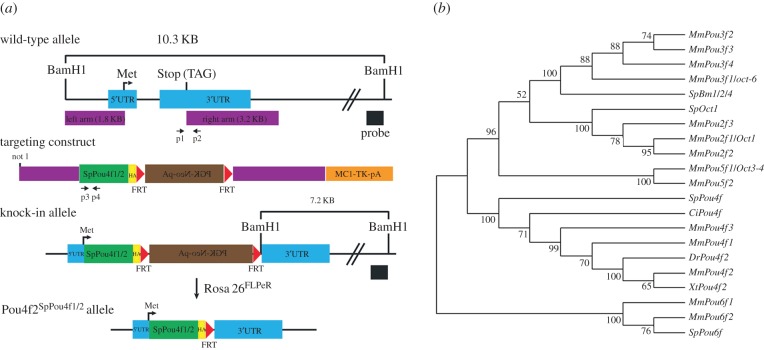
Sequence alignment of *SpPou4f1/2* and *Pou4f* and generation of the *Pou4f2^SpPou4f1/2^* KI allele. (*a*) Generation of *Pou4f2^SpPou4f1/2^* KI allele, FRT indicates FLP recombinase sites to remove the Neo selection cassette (brown box) by a *Rosa26-FLPeR* mouse line. *SpPou4f1/2* cDNA sequences (green box) were fused in frame to a HA-epitope tag (yellow box). The dark purple thick lines indicate the site of recombination into a construct carrying the *Pou4f2* coding region and upstream regulatory regon. TK (orange box) indicates the TK cassette used for negative selection. The *Bam*HI site was used for Southern genotyping, p1 and p2 are PCR primers to detect wild-type mouse *Pou4f2* allele, and p3 and p4 are primers to detect knock in *SpPou4f1/2* allele. (*b*) Phylogeny analysis of *SpPou4f1/2* and *Pou4f* genes from other organisms. The tree was constructed with MEGA software v. 5.2. using neighbour-joining method with 1000 bootstrap repeats. *Ci*, *Ciona intestinalis; Dr*, *Danio rerio; Mm*, *Mus musculus; Sp*, *Strogylocentrotus purpuratus; Xt*, *Xenopus tropicalis*.

The *SpPou4f1/2* KI allele was generated by replacing mouse *Pou4f2* with an *S. purpuratus SpPou4f1/2* cDNA sequence using recombineering. Three copies of the HA epitope tag were inserted in a frame downstream of the *SpPou4f1/2* sequence. A neomycin cassette was inserted and was flanked by two flip-recombinase target (FRT) sites. A *Bam*HI site was introduced downstream of the second FRT site for subsequent Southern genotyping (figure 1*a*).

### (c) Generation and genotyping of *Pou4f2*^*SpPou4f1/2/Z*^ and *Pou4f2*^*SpPou4f1/2/SpPou4f1/2*^ mice

The targeting construct was used to electroporate G4 129 × C57BL/6 F1 hybrid embryonic stem cells. Positive clones were selected by Southern blotting. The wild-type allele yielded a 10.3-kb band, whereas the KI allele yielded a 7.2-kb band (electronic supplementary material, figure S1*b*). The *SpPou4f1/2* KI mouse was generated by blastocyst injection. High-percentage chimeras were bred with C57BL/6 mice to generate heterozygous *Pou4f2^SpPou4f1/2/+^* KI progenies. The FRT-flanked Neo cassette in the KI progenies was further removed by a *Rosa26-FLPeR* mouse line to produce *Pou4f2^SpPou4f1/2/+^* mice [19]. *Pou4f2^+/Z^* and *Pou4f2^+/AP^* control mice were described previously [16]. *Pou4f2^SpPou4f1/2/+^* mice were then crossed with *Pou4f2^+/Z^* mice to generate *Pou4f2^SpPou4f1/2/Z^* mice. Homozygous *Pou4f2^SpPou4f1/2/SpPou4f1/2^* mice were obtained by intercrossing *Pou4f2^SpPou4f1/2/+^* mice.

PCR was used to genotype the wild-type *Pou4f2* allele and the *SpPou4f1/2* KI allele (figure 1*a*). PCR primers for the *Pou4f2* wild-type allele were p1: 5′-TCTGGAAGCCTACTTCGCCA and p2: 5′-CCGGTTCACAATCTCTCTGA. Primers to detect the *SpPou4f1/2* KI allele were p3: 5′-ATGAATATGAAGGAGCATGT and p4: 5′-TAGTTGGTGTCGTTCTTGAT.

### (d) Isolation and processing of embryos, embryonic retinas, and adult eyes, optic nerves and retinas

Embryos or adult eyes with optic nerves were harvested from different stages and processed in different ways. For histological analysis, tissues were fixed overnight in 4% paraformaldehyde (PFA) and 3% glutaradehyde in phosphate-buffered saline (PBS), subjected to PBS washing and methanol dehydration, and finally embedded in paraffin for sectioning. For immunolabelling of sections, tissues were fixed in 4% PFA for 30 min, washed three times with PBS, and embedded in optimal cutting temperature compound (Fisher Scientific).

### (e) Histology, immunolabelling and TUNEL assays of mouse samples

Haematoxylin and eosin staining was described previously [20]. Immunofluorescence staining of paraffin sections or cryosections and flat-mount staining was carried out to detect RGC axons as previously described [20]. Primary antibodies used were mouse anti-HA (Cell Signaling, 1 : 500; Cat. 2367S), goat anti-Pou4f2/Brn3 (Santa Cruz, 1 : 100; Cat. sc6026), mouse anti-Pou4f1/Brn3a (Chemicon, 1 : 400; Cat. MAB1585), mouse anti-SMI32 (Covance, 1 : 1000; Cat. SMI-32R), mouse anti-neurofilament-L (NF-L) (Invitrogen, 1 : 1000; Cat. 13-0400), chicken anti-β-galactosidase (AbCam, 1 : 2000; Cat. 9361), rabbit anti-melanopsin/Opn4 (Advanced Targeting Systems, 1 : 1000; Cat. N39), mouse anti-Islet1 (Isl-1) (DSHB, 1 : 500; Cat. 39.3F7) and rabbit anti-Tbr2/Eomes (AbCam, 1 : 1000; Cat. ab23345). The Alexa-conjugated secondary antibodies used in this study were obtained from Molecular Probes and were used at 1 : 500 dilutions. DAPI (1 µg ml^−1^, Vector Lab) was used to stain the nuclei. TUNEL assays on embryonic retinas were performed using an *in situ* cell death detection kit (Roche Applied Science) following the manufacturer's instructions. Images were acquired on an Olympus FV1000 confocal laser-scanning microscope.

### (f) Electroretinogram recordings

#### (i) Subjects

Subjects were 2.5- to 3.5-month-old mice with the following genotypes: *Pou4f2^+/+^* (*n* = 4), *Pou4f2^SpPou4f1/2/SpPou4f1/2^* (*n* = 4) and *Pou4f2^−/−^* (*n* = 4).

#### (ii) Electroretinogram recordings

Mice were dark-adapted overnight and preparations for recording were all performed under dim red illumination (*λ* > 650 nm) as previously described in [21] and the electronic supplementary material. Stimuli were provided from light-emitting diodes (*λ*_max_ = 462 nm) over a range of time-integrated flash illuminances (stimulus strengths) from −6.7 to 2.3 log scotopic (sc) cd-s m^−2^. The inter-flash interval was adjusted to allow the electroretinogram (ERG) response to return to baseline between flashes.

#### (iii) Data analysis

Amplitudes (microvolts) of a-waves were measured on the leading edge of the wave, at a fixed time (7 ms) after the brief flash, which was close to the peak amplitude for the strongest stimuli. Amplitudes of b-waves were measured between the a-wave trough and the b-wave peak after applying a low-pass 60 Hz filter to remove oscillatory potentials (the electronic supplementary material).

### (g) Expression of *SpPou4f1/2*, *Pax6* and *Opsin4* in *Strongylocentrotus purpuratus* tube foot

#### (i) Tube feet preparations

Tube feet were dissected from live *S. purpuratus* and immediately fixed in 4% PFA in PBS at room temperature for 2–4 h. After washing the collected tube feet several times in PBS, we transferred them to 100% methanol and stored them at −20°C until experimental processing.

#### (ii) *In situ* hybridization and immunohistochemistry

*SpPou4f1/2* probes were generated by cloning full-length *SpPou4f1/2* cDNA into pIRES-hrGPF-2a vector (Agilent). The *Pax6* probe has been described in Ullrich-Lüter *et al*. [10]. Both antisense- and sense-digoxigenin-labelled *SpPou4f1/2* probes were obtained using a digoxigenin-RNA labelling kit (Roche), following the manufacturer's instructions by using 1 µg of linearized plasmids. The *Pax6* RNA probe was similarly prepared using unlabelled ribonucleotides and was subsequently labelled with 2, 4-dinitrophenyl using a Label-it kit (Mirus) following the manufacturer instructions. Whole-mount *in situ* hybridization and immunostaining against Sp-Opsin4 followed the protocol of Ullrich-Lüter *et al*. [10]. Two-colour *in situ* staining was performed as described in Cole *et al*. [22]. After staining, samples were mounted in glycerol and analysed on a Leica TCS SP2 confocal laser-scanning microscope.

## 3. Results

### (a) Characterization of mature retinas in *Pou4f2^SpPou4f1/2^* mice

We inserted a full-length *SpPou4f1/2* cDNA strand containing an in-frame human influenza haemagglutinin (HA) epitope tag into the *Pou4f2* locus (figure 1*a*). Heterozygous *SpPou4f1/2* mice (*Pou4f2^SpPou4f1/2/+^*) were bred to a *Pou4f2*-null mouse line with *lacZ* inserted into the *Pou4f2* locus (*Pou4f^Z/Z^*) to generate *Pou4f2^SpPou4f1/2/Z^* offspring (electronic supplementary material, figure S1*b*). *Pou4f2^SpPou4f1/2/SpPou4f1/2^* mice were generated by intercrossing (*Pou4f2^SpPou4f1/2/+^*) heterozygotes (electronic supplementary material, figure S1*b*). *Pou4f2^+/Z^* and *Pou4f2^Z/Z^* mice served as positive and negative controls, respectively. All the mouse lines were viable and fertile. In our initial experiments, we found no qualitative or quantitative differences in the phenotypes of *Pou4f2^SpPou4f1/2/Z^* and *Pou4f2^SpPou4f1/2/SpPou4f1/2^* mice. Therefore, we used *Pou4f2^SpPou4f1/2/Z^* or *Pou4f2^SpPou4f1/2/AP^* mice for most of the reported experiments, except that the RGC anterograde tracing and ERG analysis were conducted using *wild-type* (*WT*)*, Pou4f2^SpPou4f1/2/SpPou4f1/2^* and *Pou4f2^Z/Z^* mice.

The *Pou4f2^SpPou4f1/2^* KI targeting construct contained a *PGK-neo-pA* cassette and flanking FRT sites (figure 1*a*) and *SpPou4f1/2* expression could not be detected by immunostaining using an anti-HA antibody (electronic supplementary material, figure S1*e* and data not shown). We therefore removed the *PGK-Neo-pA* cassette by breeding the *Pou4f2^SpPou4f1/2^* allele to a *Rosa26-FLPeR* mouse line. Expression from the *SpPou4f1/2* allele in E14.5 retinas of *Pou4f2^SpPou4f1/2/+^* mice, detected by immunostaining with the anti-HA antibody (electronic supplementary material, figure S1*c*), was spatially comparable with that of endogenous Pou4f2 expression (electronic supplementary material, figure S1*d*). However, expression levels in *Pou4f2^SpPou4f1/2/+^* retinas were substantially lower than those in the *Pou4f2^HA/+^* retinas using an anti-HA Pou4f2 antibody (electronic supplementary material, figure S1*f*,*g*), suggesting that SpPou4f1/2 was less stable than endogenous Pou4f2.

**Figure 2.**
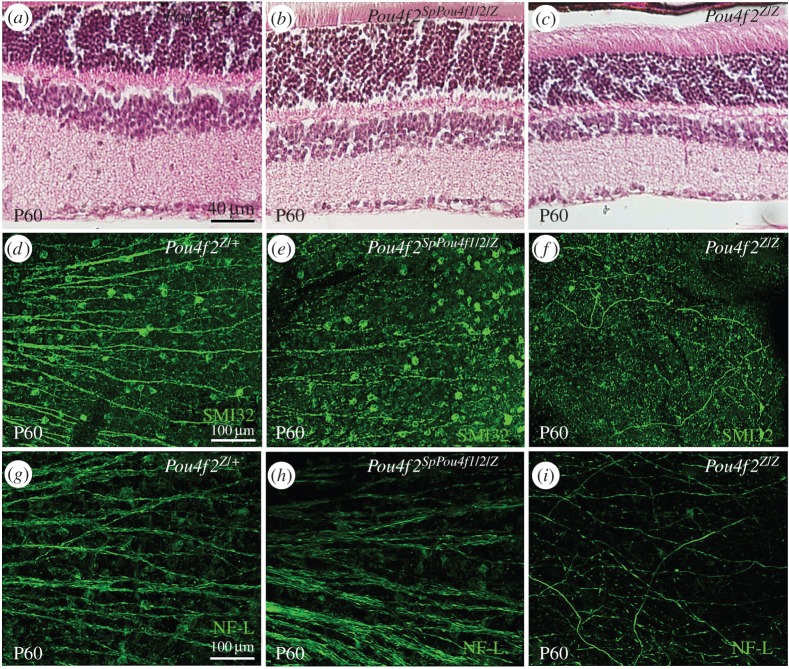
Restoration of RGCs in *Pou4f2^SpPou4f1/2/Z^* retinas. (*a–c*) H&E staining of retinas from mice at P60. (*d–f*) Immunofluorence staining of retinas from mice at P60 by anti-neurofilament heavy chain (SMI-32) antibody (green). (*g–i*) Immunofluorence staining of retinas from mice at P60 by anti-neurofilament ligh chain (NF-L) antibody (green). (*a*,*d*,*g*) *Pou4f2^Z/+^* control retinas. (*b*,*e,h*) *Pou4f2^SpPou4f1/2/Z^* retinas. (*c*,*f*,*i*) *Pou4f2^Z/Z^* retinas.

We first determined whether *SpPou4f1/2* could restore RGCs in mature adult retinas in the absence of *Pou4f2*. Retinas of *Pou4f2^Z/Z^* mice at 60 days of age (P60) lacked approximately 70% of their RGCs compared with the retinas of *wild-type* mice (*Pou4f2^+/Z^*) (figure 2*a*,*c*) [15,16]. This depletion of RGCs resulted in a thinner retina (figure 2*a*,*c*). The retinas of *Pou4f2^SpPou4f1/2/+^* mice were normal, indicating that the *SpPou4f2*-KI allele had no dominant phenotype (data not shown). Most importantly, *Pou4f2^SpPou4f1/2/Z^* retinas were not notably different from *Pou4f2^SpPou4f1/2/+^* or *Pou4f2^+/Z^* retinas (data not shown). *SpPou4f1/*2-expressing retinas appeared to have a normal ganglion cell layer with a full complement of RGCs (figure 2*b*). This result suggests that the *Pou4f2^SpPou4f1/2^* allele was able to replace *Pou4f2*'s function in forming RGCs during retinal development and in maintaining the survival of RGCs in adult retinas.

Immunostaining of flat-mounted P60 *Pou4f2^SpPou4f1/2/Z^* retinas with either SMI32 or NF-L antibodies showed the presence of many well-bundled axons emanating from *SpPou4f1/*2-expressing RGCs, whereas in *Pou4f2^Z/Z^* there was little detectable staining (figure 2*d*–*i*). The number of axons emitted from *Pou4f2^SpPou4f1/2/Z^* RGCs was substantially greater than the number observed in *Pou4f2^Z/Z^* mice (figure 2*e*,*f* and *h*,*i*), and their appearance was not qualitatively different than those of *Pou4f2^+/Z^* axons (figure 2*d*,*e* and *g*,*h*). These results suggested that *SpPou4f1/2* expression rescued the phenotype generated by the absence of *Pou4f2*. Moreover, optic nerves of *Pou4f2^SpPou4f1/2/SpPou4f1/2^* mice were indistinguishable in thickness and appearance from those of *wild-type* mice whereas *Pou4f2^Z/Z^* mice had only a thin sheath largely devoid of axon fibres (cf. arrowheads in the electronic supplementary material, figure S2*a*–*c*).

To further pursue the properties of *SpPou4f1/2*-expressing axons, we traced their path into the brain with alkaline phosphatase (AP) using a *Pou4f2-AP* allele. *Pou4f2-AP* mice were bred to *Pou4f2^+/SpPou4f1/2^* mice and retinorecepient regions in the brain were stained for AP activity. *Pou4f2^+/AP^* and *Pou4f2^SpPou4f1/2/AP^* mice each showed strong AP staining along RGC axons heavily labelling the superior colliculus (SC, the electronic supplementary material, figure S2*d*,*e*), lateral geniculate nucleus (LGN, the electronic supplementary material, figure S2*f*,*g*), suprachiasmatic nucleus (SCN, electronic supplementary material, figure S2*h*,*i*), olivary pretectal nucleus (OPN, electronic supplementary material, figure S2*j*,*k*) and accessory optic system (AOS, electronic supplementary material, figure S3), indicating that axons emanating from RGCs expressing *SpPou4f1/2* were capable of extending into and connecting with the primary visual centres in the brain. Although axonal connections were intact in AOS, we observed lower levels of AP staining. This suggested a reduction of RGC axons at the AOS.

To further inspect the functionality of these *SpPou4f1/2*-expressing RGC axons, we injected CTB-488 into the left eyes and traced their propagation into the major brain targets. We compared coronal brain sections from *wild-type* and *Pou4f2^SpPou4f1/2/SpPou4f1/2^* mice, and detected comparable CTB-488 labelling in the LGN (the electronic supplementary material, figure S2*p*,*q*), SCN (electronic supplementary material, figure S2*r*,*s*) and OPN (electronic supplementary material, figure S2*t*,*u*). The CTB-488 signal in the SC, however, was relatively weaker in *Pou4f2^SpPu4f1/2/SpPou4f1/2^* mice than that in control mice (cf. arrowheads in electronic supplementary material, figure S2*f*,*g*), yet more CTB-488 signal was retained in the retinas of *Pou4f2^SpPu4f1/2/SpPou4f1/2^* mice than in control retinas (electronic supplementary material, figure S2*l*,*m*). This difference was even more evident in the SC regions of *Pou4f2^SpPu4f1/2/Z^* and *Pou4f2^+/Z^* control mice (data not shown). These results indicated that axons emanating from RGCs expressing *SpPou4f1/2* were capable of extending into and connecting with the primary visual centres in the brain; however, they are less effective in transporting macromolecules along the path.

### (b) *SpPou4f1/2]* in the developing retina

**Figure 3.**
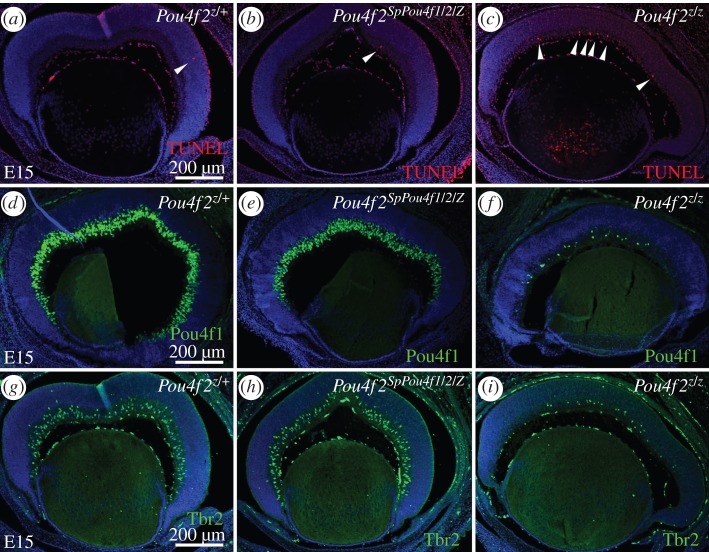
Normal RGC differentiation programme in *Pou4f2^SpPou4f1/2/Z^* retinas. TUNEL assay shows lesser cell death in retinas of *Pou4f2^Z/+^* (*a*), *Pou4f2^SpPou4f1/2/Z^* (*b*) compared with *Pou4f2^Z/Z^* (*c*). SpPou4f1/2 activates Pou4f2 downstream RGC genes Pou4f1 and Tbr2 in the absence of Pou4f2 (*d–i*). Immunostaining of retina from E15 embryos with anti-Pou4f1/Brn3a (*d–f*) and anti-Tbr2 (*g–i*).

Without Pou4f2, RGC precursors cannot perform axon related tasks including axon outgrowth, pathfinding and final targeting. Most undergo apoptosis [16]. This is probably due to a failure to activate critical genes required for RGC differentiation and survival. We compared *lacZ* expression in *Pou4f2^+/Z^*, *Pou4f2^SpPou4f1/2/Z^* and *Pou4f2^Z/Z^* E15 retinas to determine whether SpPou4f1/2 was able to generate a full complement of RGCs. *lacZ* expression in *SpPou4f1/2* retinas was indistinguishable from that in *Pou4f2* retinas; both were heavily labelled while slightly less labelling was found in *Pou4f2^Z/Z^* retinas (electronic supplementary material, figure S4*a*–*c*). TUNEL analysis showed that while many RGCs were apoptotic in the ganglion cell layer of *Pou4f2^Z/Z^* retinas (figure 3*c*), only an occasional apoptotic RGC was observed in *Pou4f2^SpPou4f1/2/Z^* retinas, which looked identical to control *Pou4f2^+/Z^* retinas (figure 3*a*,*b*). These results indicated that SpPou4f1/2 could replace Pou4f2 in sustaining RGC survival.

Several RGC-expressing genes directly activated by Pou4f2 have been identified, including another member of the Pou4f family, *Pou4f1*, which is expressed in many RGCs [23], and the T-box gene *Tbr2*/*Eomes*, whose expression is restricted to a small number of RGC subtypes [20]. Both of these genes were activated in RGCs of E15 *Pou4f2^SpPou4f1/2/Z^* retinas with expression levels equal to those of *Pou4f2^+/Z^* retinas (figure 3*d*,*e* and *g*,*h*). As expected only background expression was observed in *Pou4f2^Z/Z^* retinas (figure 3*f*,*i*). Thus, at least for *Pou4f1* and *Tbr2*, SpPou4f1/2 can perform the necessary functions for binding to Pou4f consensus sites in the *cis*-regulatory regions of these genes and activating gene expression.

To quantify the extent to which SpPou4f1/2 could rescue the RGC differentiation in mature retinas, *Pou4f1* and *Tbr2* were chosen for the analysis because their expression is mutually exclusive [24]. *Pou4f1* is expressed in approximately 70–75% of RGCs and *Tbr2* is expressed in approximately 15–20%. *Tbr2* regulates the formation and maintenance of *Opn4*-expressing intrinsic photosensitive RGCs (ipRGCs) [24,25]. By contrast, Pou4f1 expression marks distinct subtypes of RGCs [26]. Another essential RGC gene, *Isl1*, which is widely expressed in RGCs [27], has an expression pattern partially overlapping with that of *Pou4f2*. The precise timing and spatial expression patterns of *Pou4f1* and *Isl1* are required for the normal development of RGCs [26,27], and the expression of *Tbr2* and *Opn4* are indicators of a conserved *Pou4f2-Tbr2-Opn4* genetic regulatory cascade as well as regulatory cascades for other *Tbr2*-expressing RGC subtypes [24]. We asked whether *SpPou4f1/2* was able to reproduce this spatio-temporal programme in mature retinas in the absence of *Pou4f2*.

This turned out to be the case. Double immunostaining P30 *SpPou4f1/2*-expressing retinas for Pou4f1 and Tbr2 showed the expected pattern of staining (electronic supplementary material, figure S5*b*). This pattern was very similar to that of control P30 retinas (electronic supplementary material, figure S5*a*).

*SpPou4f1/2*-expressing retinas co-immunostained with Opn4 and Isl1 also showed spatial expression patterns that were identical to those of *wild-type* mice (electronic supplementary material, figure S5*c*,*d*). As expected only a minor fraction of *Isl1*-expressing RGCs were co-expressed with Opn4. Notably, Opn4 staining reveals *Opn4*-expressing RGCs extending an extensive meshwork of neurite processes. These were readily observed in both *Pou4f2^+/Z^* and *Pou4f2^SpPou4f1/2/Z^* retinas (electronic supplementary material, figure S5*c*,*d*).

### (c) Electroretinogram responses for *Pou4f2^SpPou4f1/2/SpPou4f1/2^* mice

**Figure 4.**
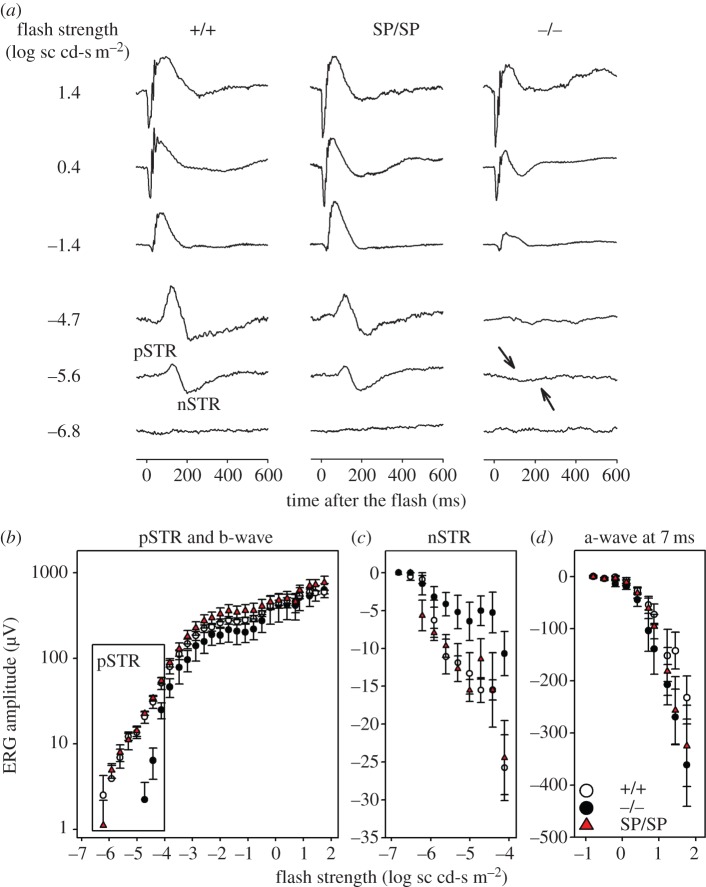
ERG recordings of *Pou4f2^SpPou4f1/2/SpPou4f1/2^* retinas. (*a*) Scotopic full-field flash ERG responses recorded from one mouse in each of the three genotypes. From left to right, *Pou4f2^+/+^* (+/+), *Pou4f2^SpPou4f1/2/SpPou4f1/2^* (SP/SP), *Pou4f2^−/−^* (−/−). Stimulus strength increases from bottom to top. Arrows in the right column indicated missing STRs in this animal. (*b–d*) Stimulus versus ERG amplitude plots measured for the three genotypes. *Pou4f2^+/+^*(+/+; *n* = 4), *Pou4f2^SpPou4f1/2/SpPou4f1/2^* (Sp/Sp; *n* = 4), *Pou4f2^−/−^* (−/−; *n* = 4). (*b*) pSTR (box) and b-wave amplitudes. (*c*) nSTR amplitudes and (*d*) a-wave amplitudes. The nSTR amplitudes saturated around −4.1 log sc cd-s m^−2^, and then a larger negative wave of unknown origin emerged. The error bars are standard errors.

In *Pou4f2^+/+^* mice, the dark-adapted ERG in response to weak stimuli, e.g. was dominated by two signals from the inner retina, positive (p) and negative (n) scotopic threshold responses (STR) [21] (figure 4*a*). The very sensitive positive STR, present at stimulus strengths lower than those for which the b-wave contributes to the positive peak, relies essentially entirely on the integrity of RGCs for its generation [21,28]. The negative STR amplitude is also impacted by the loss of RGCs, but the reported extent of loss has varied [28]. *Pou4f2^−/−^* mice lacked both waves originating from the inner retina that were present in the other genotypes (figure 4*a*).

The ERG responses to the weakest stimuli in *Pou4f2^SpPou4f1/2/SpPou4f1/2^* mice (figure 4*a*) were much more similar to those in *Pou4f2^+/+^* mice than to those in *Pou4f2^−/−^* mice, indicating the presence of RGC function. In all three groups, the ERG responses to stronger stimuli, e.g. −1.4 log sc cd-s m^−2^, were similar: the b-wave, thought to be generated by rod-driven bipolar cells increased in amplitude with stimulus strength, and for the strongest stimuli, e.g. 1.4 log sc cd-s m^−2^, a negative-going a-wave, which reflects photoreceptor currents, was present at the beginning of the response [29].

To quantify the ERG responses, stimulus versus ERG amplitude plots were constructed based on measurements of the ERG waves, pSTR and b-wave, measured at the peak (figure 4*b*) and trough of the response (figure 4*c*), respectively. Figure 4*d* shows the amplitude measured at 7 ms after the flash on the leading edge of the a-wave. As predicted by the ERG traces (figure 4*a*; electronic supplementary material, table S1), the pSTR amplitudes were significantly lower in the *Pou4f2^−/−^* mice than in the other groups. For pSTR, amplitudes for the different groups were compared only in the boxed region in the pSTR/b-wave plot. The nSTR amplitudes of the *Pou4f2^−/−^* mice were also significantly lower than in *Pou4f2^SpPou4f1/2/SpPou4f1/2^* mice, and just missed being significantly lower the *Pou4f2^+/+^* mice, whereas p- and nSTRs in *Pou4f2^+/+^* and *Pou4f2^SpPou4f1/2/SpPou4f1/^* mice were not significantly different from each another. No other ERG amplitude measures were significantly different across the groups, although the b-wave amplitudes for the *Pou4f2^−/−^* mice tended to be lower than those for the other groups. Implicit times for measured responses also did not differ significantly across groups.

### (d) *SpPou4f1/2* expression in *Strongylocentrotus purpuratus* tube feet

Our results suggest that *SpPou4f1/2* performs similar regulatory functions to those of *Pou4f2* in photoreceptor neurons of *S. purpuratus* tube feet. It is also likely that *SpPou4f1/2* is regulated directly or indirectly by the *S. purpuratus* orthologue of *Pax6*, as *Pax6* is expressed in neuronal cells of the tube feet disc [9,10]. Our expectation was that *SpPou4f1/2* would be expressed in tube feet photoreceptor cells and its expression would overlap with that of both *Pax6* and the sea urchin photopigment *Opsin4* [10]. Accordingly, we performed *in situ* hybridization with adult *S. purpuratus* tube feet using *SpPou4f1/2* and *Pax6* RNA probes and immunolabelling using an anti-Opsin4 antibody [10].

**Figure 5.**
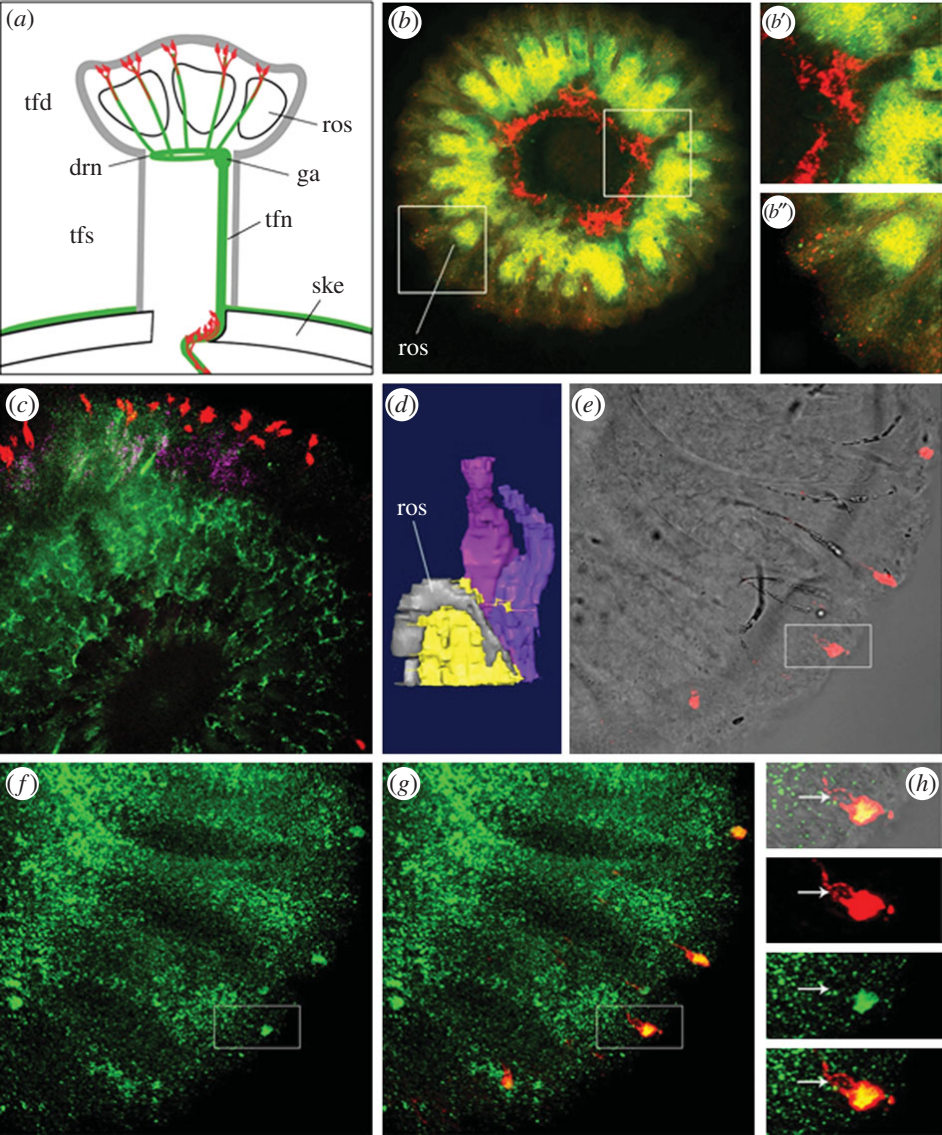
Co-expression analysis of SpPou4f1/2, SpPax6 and SpOpsin4 in *S. purpuratus* tube feet. (*a*) Schematic view of a sea urchin tube feet displaying the morphology of tube feet stalk (tfs), disc (tfd) and rosette (ros); the nervous system (green) is represented by tube feet nerve (tfn), ganglion (ga) and disc ring nerve (drn). Sp-Opsin4 positive photoreceptor cells (red) are located at the rim of the disc and in a depression of the skeleton (ske) at the base of the tube feet. (*b*,*c*,*e*,*f*,*g*,*h*) Z-stack projection of tube feet disc. (*b*) Bottom view (seen from the stalk), (*c*,*e*,*f*,*g*,*h*) top view, showing *SpPou4f1/2* and *SpPax6* mRNA expression and Sp-Opsin4 protein in disc photoreceptors. The whole tube feet disc is shown in (*b*) while (*c*), (*e*–*g*) display only a quadrant of it. *SpPou4f1/2* mRNA is shown in yellow/green (*b*) or green (*c*,*f*–*h*), *SpPax6* mRNA in red (*b*) or purple (*c*), SpOpsin4 protein in red (*c*,*e*,*g* and *h*). (*b*) *SpPou4f1/2* mRNA expression associated with skeletal rosettes, *SpPax6* mRNA in stalk and disc. (*b*′ and *b*″) are details of (*b*) as indicated. (*c*) Expression of *SpPou4f1/2* corresponds to neuronal tissue and axonal projection area of photoreceptors. *SpPax6* expression in the area between skeletal rosette elements correlates with localization of photoreceptor cell bodies and SpOpsin4 protein in the apical part of the photoreceptor cells. (*d*) Three-dimensional reconstruction of serial TEM sections clarifies cell position and identity in *SpPou4f1/2*, *SpPax6* and SpOpsin4 expression regions. Skeletal rosette (grey) with associated nerve tissue (yellow) and two photoreceptors (purple and blue). (*e*–*g*) Different overlays showing coexpression of *SpPou4f1/2* mRNA and SpOpsin4 protein in the apical region of photoreceptors. (*h*) A twofold zoom of the area indicated by the rectangle in (*e*–*g*) showing a single photoreceptor (arrow shows nucleus).

*SpPou4f1/2* transcripts were detected in neuronal tissue associated with the tube feet disc skeletal rosettes (figure 5*b,c,e–h*). The *SpPou4f1/2* staining pattern viewed from the top of the tube feet resembled that of an antibody against *S. purpuratus* synaptotagmin, which labels neuronal processes [8] (figure 5*b,c*). When viewed from the stalk side of the disc (figure 5*b*), *SpPou4f1/2* expression was far more intense, as the majority of the nerve fibres lies on this side and enclose the skeleton from underneath, a finding confirmed by previous electron microscopic studies [10]. *SpPou4f1/2* sense control showed no detectable signal over background (electronic supplementary material, figure S6). From *SpPou4f1/2* expression, a more precise location of the previously reported expression of *Pax6* could also be obtained [10]. Comparison of the two expression patterns showed that *Pax6* was expressed in proximity to, but not overlapping with, *SpPou4f1/2* expression (figure 5*b*,*c*). Probably due to reported differences in expression quantities between the tube feet stalk and the disc [9,12] (figure 5*b*,*c*), single-cell resolution could not be obtained for *Pax6* expression in the tube feet disc.

A TEM three-dimensional reconstruction of serial sections of a tube feet disc illustrates cell position and identity in the area of *SpPou4f1/2*, *Pax6* and Opsin4 localization (figure 5*d*). The distal portion of a disc rosette is accompanied by a massive nerve tissue. Two photoreceptor cells are adjacent to and above the large nerve tissue, which send cell extensions into it [10]. *SpPou4f1/2* expression can be clearly correlated to the massive nerve tissue, while *Pax6* expression correlates with the region where the cell bodies of photoreceptors are localized. Higher magnification reveals that *SpPou4f1/2* expression colocalizes with Opsin4 protein in the same cells (figure 5f*–h*). While Opsin4 protein was localized in a portion of the photoreceptor cell bodies and in their dendrites, which contact the epidermal surface (figure 5*e*,*g* and *h*), *SpPou4f1/2* expression is restricted to a smaller area of the photoreceptor cell body, close to the nucleus.

These results showed that *SpPou4f1/2* mRNA and Opsin4 protein colocalized within the same photoreceptor cells. However, our results suggested that *SpPou4f1/2* and *Pax6* transcripts were not necessarily expressed within the same photoreceptor cell or alternatively that they were segregated into distinct domains within the same photoreceptor cell. Pax6 and SpPou4f1/2 are transcription factors that are largely localized to the cell nucleus. The non-overlapping or partially overlapping expression of *Pax6* and *SpPou4f1/2* transcripts might suggest that the two proteins are being translated in distinct regions of tube feet photoreceptor cells, or that *Pax6* expression is not persistent in fully differentiated *S. purpuratus* Opsin4 positive photoreceptor cells.

## 4. Discussion

Our results indicate that *SpPou4f1/2* can substitute for *Pou4f2* and that there is a high degree of functional conservation between the two genes. In contrast with the much later role that *Pou4f2* plays in RGC differentiation, *Pax6* sits at the top of the hierarchical tier for eye development, setting up the eye field and activating a highly conserved gene regulatory network [30,31]. The subsequent processes that lead to the formation of the lens, retina and other tissues of the eye are regulated by transcription factor networks downstream of the *Pax6* network. Our results support the notion that many genes downstream of *Pou4f2* and *SpPou4f1/2* are held in common.

Although *Pou4f2* and *SpPou4f1/2* share high sequence similarity in their Pou-specific and Pou-homeodomains, there is only weak similarity outside of these domains. The bipartite DNA-binding domains of POU domain factors confer versatility by flexible interactions with their DNA target sites [32]. Our finding that SpPou4f1/2 binds to DNA at Pou4f2 binding sites and activates many or all the genes that are activated by Pou4f2 may not be surprising, given the high degree of sequence similarity in the DNA-binding domains of the two proteins. However, POU domain transcription factors must interact with complex transcriptional machineries in order to function [33]. Several proteins are known to interact with POU domain factors at sequences mapping outside of the bipartite DNA-binding domains [33,34]. These interactions provide further functional specificity to target gene selection. SpPou4f1/2 is probably to interact with a multitude of proteins in tube feet photosensory neurons. The observed functional equivalence of *Pou4f2* and *SpPou4f1/2* implies that their interactions with co-activators, co-repressors and other components of the transcriptional machinery are also functionally conserved. Given the high degree of sequence divergence of Pou4f2 and SpPou4f1 outside of their Pou-specific and Pou-homeodomain, interactions with other proteins are likely to be confined to these conserved domains. However, the lack of sequence similarity outside the Pou-specific and Pou-homeodomains does not preclude a conserved role for at least some amino acids in these regions. We think it is unlikely that a Pou4f class protein with sequences chosen at random outside the conserved domains would properly fold into a functional protein.

Our experiments support the view of functional equivalence of the Pou4f factors. While many genes expressed in photosensitive neurons in the tube feet and RGCs in the retina are likely to be held in common, there are likely to be genes specifically required for the specialized neurons of each species. Tube feet-specific genes might have acquired consensus Pou4f DNA-binding sites at some point during echinoderm evolution. The corresponding orthologous genes in the mouse genome would not have Pou4f DNA-binding sites and would not be expressed in RGCs. Conversely, for genes specifically expressed in RGC differentiation, there would be a set of corresponding orthologous genes in the *S. purpuratus* genome that would not have SpPou4f1/2 DNA-binding sites.

Low levels of *SpPou4f1/2* expression in the developing retina were not surprising as its expression is under the control of the relatively weak *Pou4f2* promoter and intron 1 was deleted in the KI allele. Nevertheless, the low expression of *SpPou4f1/2* was sufficient to form fully functional RGCs, even in the complete absence of *Pou4f2*. *Pou4f2^Z/Z^* mice are genetically null and Pou4f2 protein cannot be detected in mutant retinas [15,16]. However, *Pou4f2^+/Z^* mice are phenotypically *wild-type*. As *Pou4f2^SpPou4f1/2/Z^* expression levels are significantly lower than *Pou4f2^+/Z^* expression in the developing retina, the threshold level for Pou4f2 necessary to function in RGC development is probably to be substantially lower.

## Supplementary Material

Supplementary Figures

## Data accessibility

The *Pou4f2^SpPou4f1/2^* mouse line generated in this study is freely available to all investigators upon request as stipulated in MD Anderson's Memorandum Transfer Agreement policies.

## References

[1] Gehring WJ 2002 The genetic control of eye development and its implications for the evolution of the various eye-types. Int. J. Dev. Biol. 46, 65–73. 11902689

[2] Peter IS, Davidson EH 2011 Evolution of gene regulatory networks controlling body plan development. Cell 144, 970–985. (10.1016/j.cell.2011.02.017) 21414487 PMC3076009

[3] Popodi E, Raff RA 2001 Hox genes in a pentameral animal. BioEssays 23, 211–214. (10.1002/1521-1878(200103)23:3<211::AID-BIES1030>3.0.CO;2-6) 11223877

[4] Millott N 1954 Sensitivity to light and the reactions to changes in light intensity of the echinoid, *Diadema antillarum* Philippi. Phil. Trans. R. Soc. Lond. B 238, 187–202. (10.1098/rstb.1954.0009)

[5] Blevins E, Johnsen S 2004 Spatial vision in the echinoid genus *Echinometra*. J. Exp. Biol. 207, 4249–4253. (10.1242/jeb.01286) 15531646

[6] Millott N 1966 Coordination of spine movements in echinoids. In Physiology of Echinodermata, pp. 187–220. New York, NY: Interscience.

[7] Yoshida 1966 Photosensitivity. In Physiology of Echinodermata *(ed*. Boolootian RA), pp. 435–464. New York, NY: Interscience.

[8] Burke RD, et al. 2006 A genomic view of the sea urchin nervous system. Dev. Biol. 300, 434–460. (10.1016/j.ydbio.2006.08.007) 16965768 PMC1950334

[9] Agca C, Elhajj MC, Klein WH, Venuti JM 2011 Neurosensory and neuromuscular organization in tube feet of the sea urchin *Strongylocentrotus purpuratus*. J. Comp. Neurol. 519, 3566–3579. (10.1002/cne.22724) 21800307

[10] Ullrich-Luter EM, Dupont S, Arboleda E, Hausen H, Arnone MI 2011 Unique system of photoreceptors in sea urchin tube feet. Proc. Natl Acad. Sci. USA 108, 8367–8372. (10.1073/pnas.1018495108) 21536888 PMC3100952

[11] Lesser MP, Carleton KL, Bottger SA, Barry TM, Walker CW 2011 Sea urchin tube feet are photosensory organs that express a rhabdomeric-like opsin and PAX6. Proc. R. Soc. B 278, 3371–3379. (10.1098/rspb.2011.0336) PMC3177635 21450733

[12] Czerny T, Busslinger M 1995 DNA-binding and transactivation properties of Pax-6: three amino acids in the paired domain are responsible for the different sequence recognition of Pax-6 and BSAP (Pax-5). Mol. Cell Biol. 15, 2858–2871. (10.1128/MCB.15.5.2858) 7739566 PMC230517

[13] Raible F, Tessmar-Raible K, Arboleda E, Kaller T, Bork P, Arendt D, Arnone MI 2006 Opsins and clusters of sensory G-protein-coupled receptors in the sea urchin genome. Dev. Biol. 300, 461–475. (10.1016/j.ydbio.2006.08.070) 17067569

[14] Erkman L et al. 1996 Role of transcription factors Brn-3.1 and Brn-3.2 in auditory and visual system development. Nature 381, 603–606. (10.1038/381603a0) 8637595

[15] Gan L, Xiang M, Zhou L, Wagner DS, Klein WH, Nathans J 1996 POU domain factor Brn-3b is required for the development of a large set of retinal ganglion cells. Proc. Natl Acad. Sci. USA 93, 3920–3925. (10.1073/pnas.93.9.3920) 8632990 PMC39460

[16] Gan L, Wang SW, Huang Z, Klein WH 1999 POU domain factor Brn-3b is essential for retinal ganglion cell differentiation and survival but not for initial cell fate specification. Dev. Biol. 210, 469–480. (10.1006/dbio.1999.9280) 10357904

[17] Tamura K, Peterson D, Peterson N, Stecher G, Nei M, Kumar S 2011 MEGA5: molecular evolutionary genetics analysis using maximum likelihood, evolutionary distance, and maximum parsimony methods. Mol. Biol. Evol. 28, 2731–2739. (10.1093/molbev/msr121) 21546353 PMC3203626

[18] Gold DA, Gates RD, Jacobs DK 2014 The early expansion and evolutionary dynamics of POU class genes. Mol. Biol. Evol. 31, 3136–3147. (10.1093/molbev/msu243) 25261405 PMC4245813

[19] Farley FW, Soriano P, Steffen LS, Dymecki SM 2000 Widespread recombinase expression using FLPeR (Flipper) mice. Genesis 28, 106–110. (10.1002/1526-968X(200011/12)28:3/4<106::AID-GENE30>3.0.CO;2-T) 11105051

[20] Mao CA, Kiyama T, Pan P, Furuta Y, Hadjantonakis AK, Klein WH 2008 Eomesodermin, a target gene of Pou4f2, is required for retinal ganglion cell and optic nerve development in the mouse. Development 135, 271–280. (10.1242/dev.009688) 18077589 PMC2893890

[21] Saszik SM, Robson JG, Frishman LJ 2002 The scotopic threshold response of the dark-adapted electroretinogram of the mouse. J. Physiol. 543, 899–916. (10.1113/jphysiol.2002.019703) 12231647 PMC2290546

[22] Cole AG, Rizzo F, Martinez P, Fernandez-Serra M, Arnone MI 2009 Two ParaHox genes, *SpLox* and *SpCdx*, interact to partition the posterior endoderm in the formation of a functional gut. Development 136, 541–549. (10.1242/dev.029959) 19144720

[23] Trieu M, Rhee JM, Fedtsova N, Turner EE 1999 Autoregulatory sequences are revealed by complex stability screening of the mouse Brn-3.0 locus. J. Neurosci. 19, 6549–6558. 10414983 10.1523/JNEUROSCI.19-15-06549.1999 PMC6782789

[24] Sweeney NT, Tierney H, Feldheim DA 2014 Tbr2 is required to generate a neural circuit mediating the pupillary light reflex. J. Neurosci. 34, 5447–5453. (10.1523/JNEUROSCI.0035-14.2014) 24741035 PMC3988404

[25] Mao CA, Li H, Zhang Z, Kiyama T, Panda S, Hattar S, Ribelayga CP, Mills SL, Wang SW 2014 T-box transcription regulator *Tbr2* is essential for the formation and maintenance of Opn4/melanopsin-expressing intrinsically photosensitive retinal ganglion cells. J. Neurosci. 34, 13 083–13 095. (10.1523/JNEUROSCI.1027-14.2014) PMC4172803 25253855

[26] Badea TC, Cahill H, Ecker J, Hattar S, Nathans J 2009 Distinct roles of transcription factors Brn3a and Brn3b in controlling the development, morphology, and function of retinal ganglion cells. Neuron 61, 852–864. (10.1016/j.neuron.2009.01.020) 19323995 PMC2679215

[27] Mu X, Fu X, Beremand PD, Thomas TL, Klein WH 2008 Gene regulation logic in retinal ganglion cell development: Isl1 defines a critical branch distinct from but overlapping with Pou4f2. Proc. Natl Acad. Sci. USA 105, 6942–6947. (10.1073/pnas.0802627105) 18460603 PMC2383966

[28] Smith BJ, Wang X, Chauhan BC, Cote PD, Tremblay F 2014 Contribution of retinal ganglion cells to the mouse electroretinogram. Doc. Ophthalmol. 128, 155–168. (10.1007/s10633-014-9433-2) 24659322

[29] Robson JG, Frishman LJ 2014 The rod-driven a-wave of the dark-adapted mammalian electroretinogram. Prog. Retinal Eye Res. 39, 1–22. (10.1016/j.preteyeres.2013.12.003) PMC3939025 24355774

[30] Treisman JE 1999 A conserved blueprint for the eye? BioEssays 21, 843–850. (10.1002/(SICI)1521-1878(199910)21:10<843::AID-BIES6>3.0.CO;2-J) 10497334

[31] Silver SJ, Rebay I 2005 Signaling circuitries in development: insights from the retinal determination gene network. Development 132, 3–13. (10.1242/dev.01539) 15590745

[32] Phillips K, Luisi B 2000 The virtuoso of versatility: POU proteins that flex to fit. J. Mol. Biol. 302, 1023–1039. (10.1006/jmbi.2000.4107) 11183772

[33] Andersen B, Rosenfeld MG 2001 POU domain factors in the neuroendocrine system: lessons from developmental biology provide insights into human disease. Endocr. Rev. 22, 2–35. (10.1210/edrv.22.1.0421) 11159814

[34] Gonzalez MM, Carlberg C 2002 Cross-repression, a functional consequence of the physical interaction of non-liganded nuclear receptors and POU domain transcription factors. J. Biol. Chem. 277, 18 501–18 509. (10.1074/jbc.M200205200) 11891224

