# Supplementary material for: Substituting mouse transcription factor Pou4f2 with a sea urchin orthologue restores retinal ganglion cell development
Source: Proc Biol Sci. 2016 Mar 16;283(1826):20152978. doi: 10.1098/rspb.2015.2978 (PMC4810862; doi:10.1098/rspb.2015.2978)
Supplement: Supplementary Figures [file rspb20152978supp1.pdf]

Supplementary Tables

Suppl. Table 1.

ERG wave	Range<br>Log sc cd-s/m^2	F value	P values	*Pou4f*$^{-/-}$ *vs* *Pou4f*$^{+/+}$	+/+ *vs* SP/SP	-/- *vs* SP/SP
a-wave	0.1 to 2.3	0.754	0.574			
b-wave	-4.1 to 2.3	0.445	0.812			
nSTR	-6.5 to -4.4	2.28	0.035*	0.07	0.996	0.05*
pSTR	-6.5 to -4.4	12.521	0.000002*	0.001*	0.945	0.001*

*P<0.05

Suppl. Table 1. Amplitudes of scotopic ERG waves in Pou4f2 $^{SpPou4f1/2/SpPou4f1/2}$ mice

pSTR amplitudes were significantly lower in the *Pou4f2*$^{-/-}$ mice than in the other groups. P<0.05 was considered to be significant. *Pou4f2*$^{+/+}$: +/+, *Pou4f2*$^{SpPou4f1/2/SpPou4f1/2}$: SP/SP, *Pou4f2*$^{-/-}$: -/-.

Supplementary Figures

Suppl. Figure 1

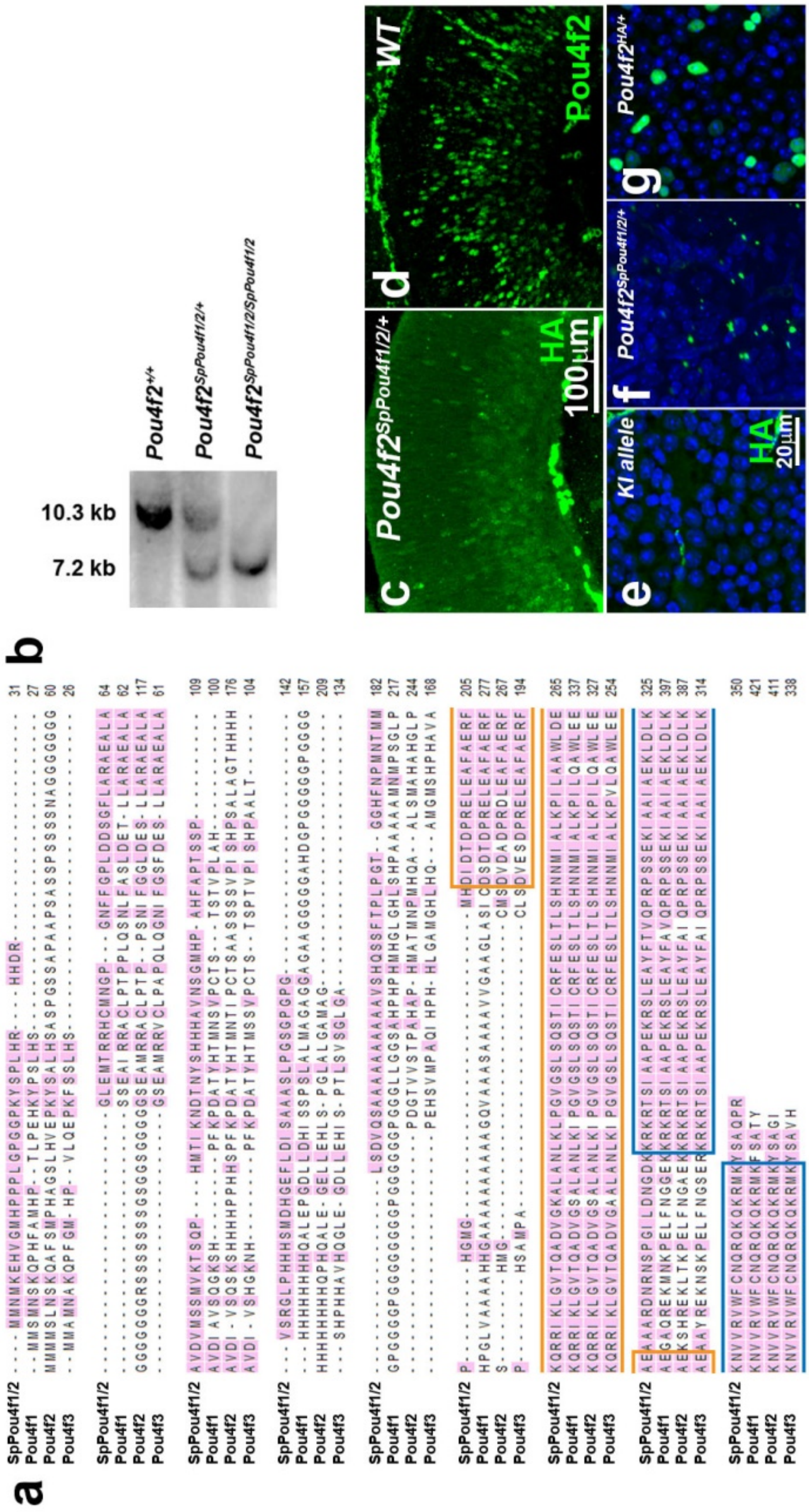

Suppl. Figure 1. Sequence alignment of *SpPou4f1/2* and *Pou4f* and expression of *SpPou4f1/2* in *Pou4f2*$^{SpPou4f1/2}$ knock-in allele.

(*a*) Amino acid sequence comparisons of mouse and *S. purpuratus Pou4f* genes. Dashed lines indicate absence of amino acids, sequences boxed in orange and blue indicate POU specific domain and POU homeobox, respectively. (*b*) Southern genotyping using a 3′ probe to distinguish a 10.3 kb *Bam*HI wild-type *Pou4f2* fragment from a 7.2 kb *Bam*HI *Pou4f2*$^{SpPou4f1/2}$ fragment. (*c, d*) Expression of *SpPou4f1/2* from the *Pou4f2* locus at E14. Retinal sections from E14 immuno-labeled with anti-HA antibody (*c*) and wild-type labeled with anti-Pou4f2 antibody (*d*). (*e,f,g*) Flat-mounted retinas immuno-labeled with anti-HA showing expression of *SpPou4f1/2* from the *Pou4f2* locus at P30 knock-in animal (*e*, before Neo-cassette is removed), *Pou4f2*$^{SpPou4f1/+}$ heterozygous animal (*f*, Neo cassette has been removed by *Rosa26*^{FLPeR}). (*g*) A flat-mounted *Pou4f2*$^{HA/+}$ retina (control) immuno-labeled with anti-HA showing robust expression of pou4f2HA fusion protein [1].

Suppl Figure 2.

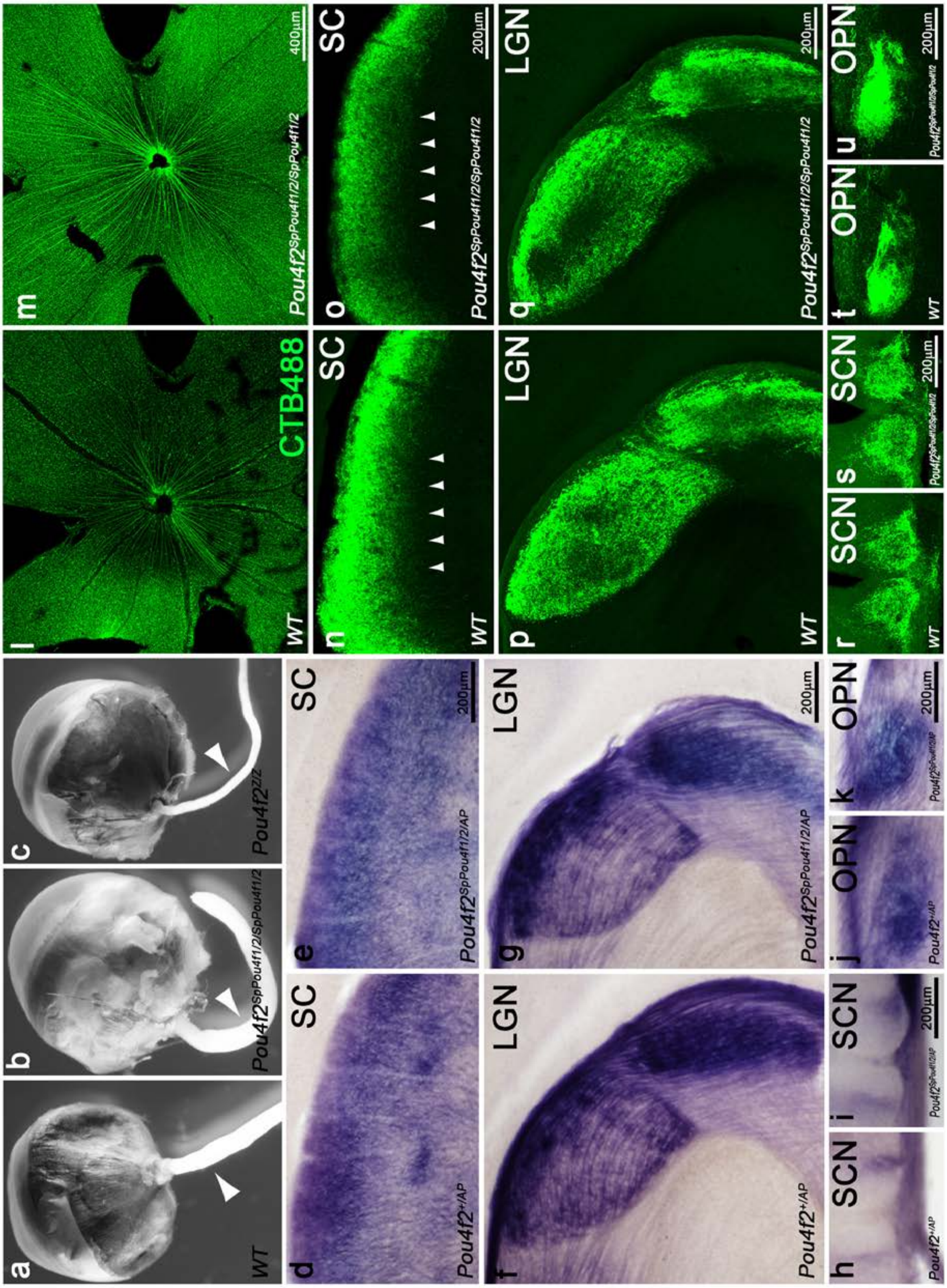

Suppl Figure 2. Normal anatomy with subtle aberrant functionality in dye transferring in optic nerves of *Pou4f2*$^{SpPou4f1/2/SpPou4f1/2}$ mice. Eyes and optic nerves from *WT* (*a*), *Pou4f2*$^{SpPou4f1/2/SpPou4f1/2}$ (*b*), *Pou4f2*$^{Z/Z}$ (*c*) mice. (*d-k*) Images showing the alkaline phosphatase staining for SpPou4f1/2-expressing RGC axons in SC (*d, e*), LGN (*f, g*), SCN (*h, i*), and SC (*j, k*) regions of the brains of *Pou4f2*$^{+/AP}$ (*d, f, h, j*) and *Pou4f2*$^{SpPou4f1/2/AP}$ (*e, g, i, k*) mice. (*l-u*) Confocal images of anterograde tracing using CTB-488 for RGC axon projections in the retinas (*l, m*), SC (*n, o*), LGN (*p, q*), SCN (*r, s*), and SC (*t, u*) of *WT* (*l, n, p, r, t*) and *Pou4f2*$^{SpPou4f1/2/SpPou4f1/2}$ (*m, o, q, s, u*) mice. SC: superior colliculus. LGN: lateral geniculate nuclei. SCN: suprachiasmatic nuclei. OPN: olivary pretectal nuclei.

Suppl Figure 3.

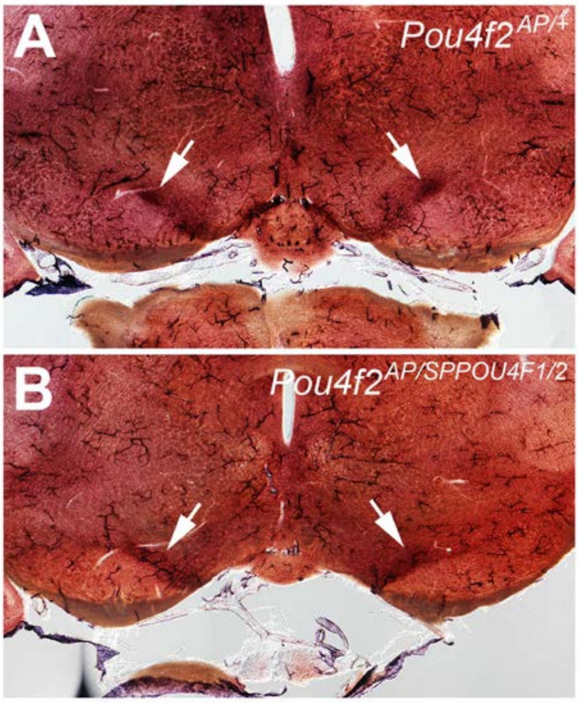

Suppl Figure 3. RGC axon projections in AOS of *Pou4f2*$^{SpPou4f1/2/AP}$ mice. Alkaline phosphatase staining for AOS of *Pou4f2*$^{SpPou4f1/2/AP}$ mice (B) was compared to control *Pou4f2*$^{+/AP}$ mice (A). Both mice had RGC axons in AOS confirmed by AP staining. *Pou4f2*$^{SpPou4f1/2/AP}$ mice had relatively lower AP levels at AOS. AOS: accessory optic system.

Suppl. Figure 4

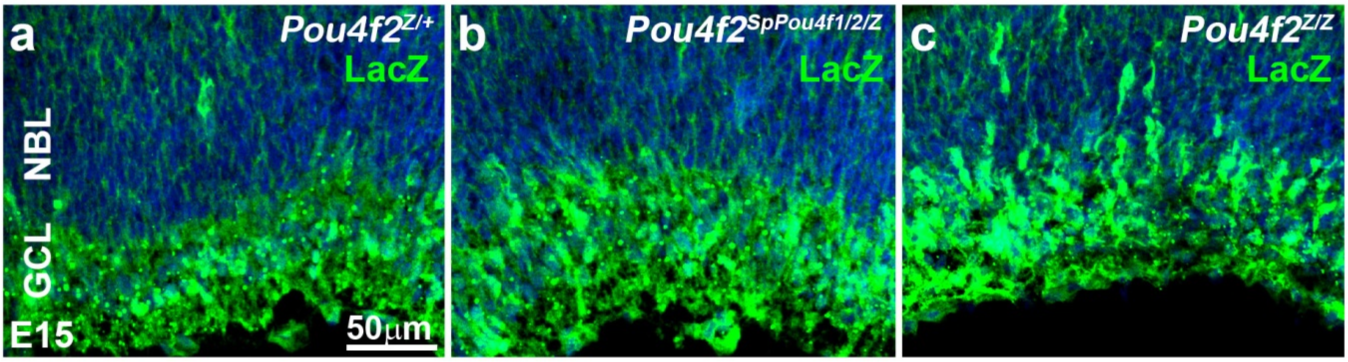

Suppl. Figure 4. Expression of *LacZ* in *Pou4f2* knock-in alleles.

Immunostaining of anti-β-galactosidase antibody in retinal sections of *Pou4f2*$^{Z/+}$ (*a*), *Pou4f2*$^{SpPou4f1/2/Z}$ (*b*), *Pou4f2*$^{Z/Z}$ (*c*).

Suppl. Figure 5.

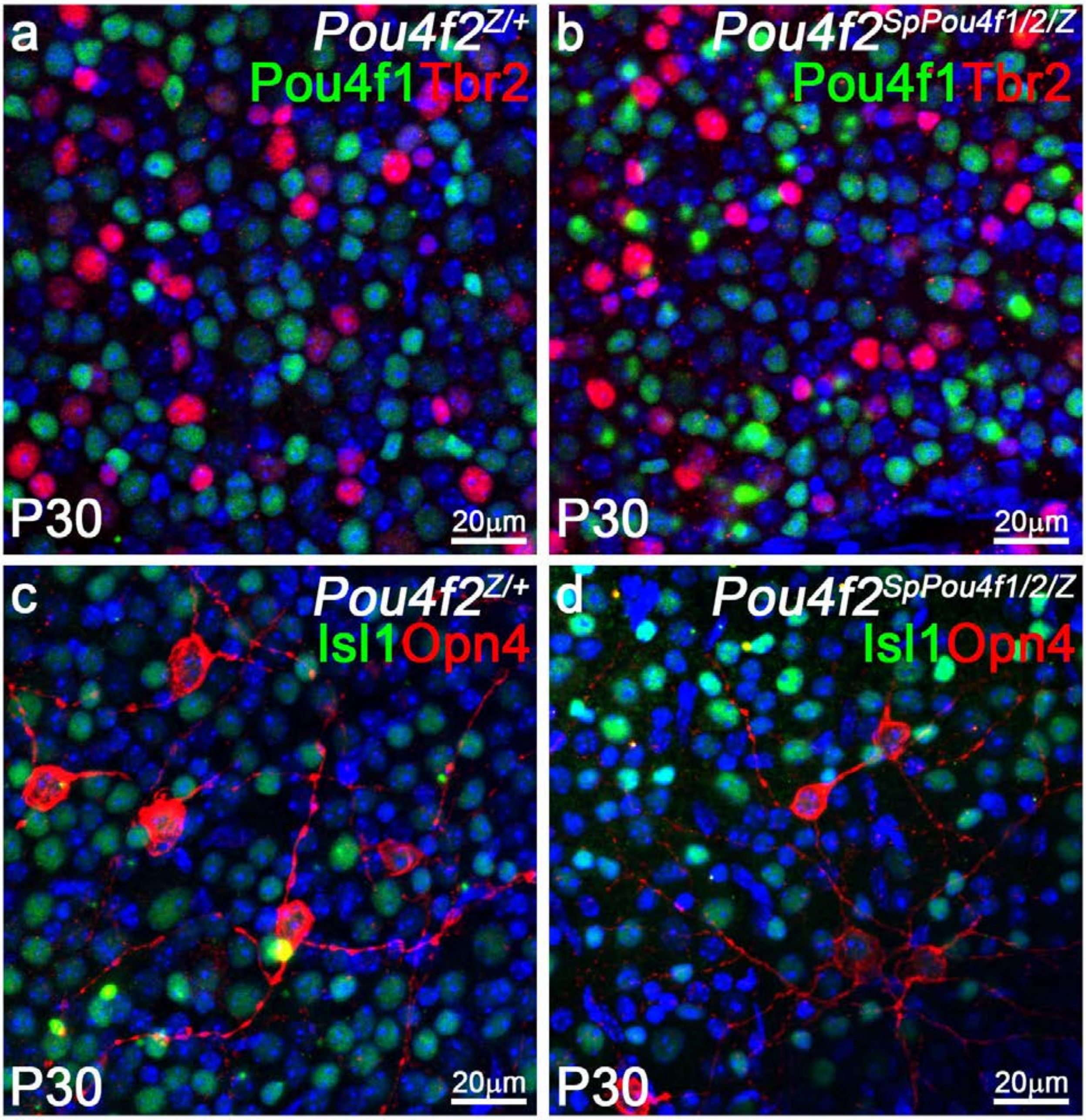

Suppl. Figure 5. Normal expression of RGC genes in adult retinas of *Pou4f2*$^{SpPou4f1/2}$ mice. (a-d) Flat-mounted P30 retinas from *Pou4f2*$^{Z/+}$ (*a, c*) and *Pou4f2*$^{SpPou4f1/2/Z}$ (*b, d*) mice co-labelled with *(a, b*) anti-Pou4f1 (green) and anti-Tbr2 (red) antibodies, and (*c, d*) anti-Isl1 (green) and anti-Opn4 (red) antibodies. Blue is DAPI staining for nuclei.

Suppl. Figure 6.

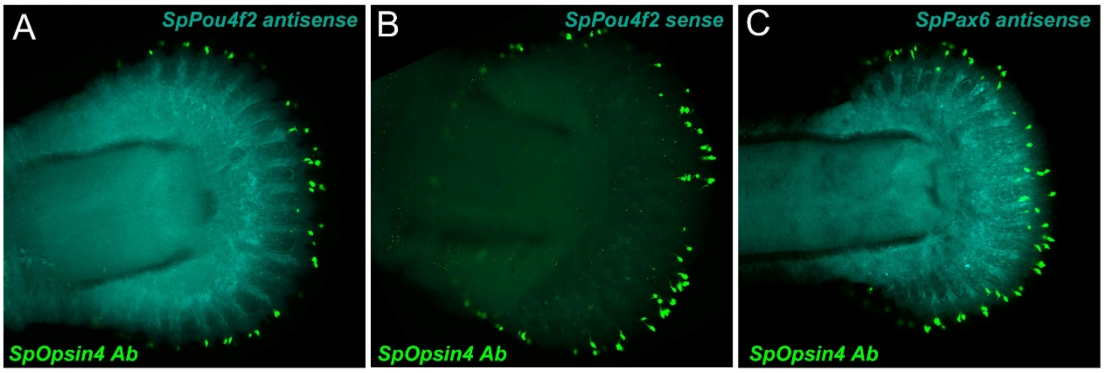

Suppl. Figure 6. ***Controls for SpPou4f2, SpPax6 and SpOpsin4 co-expression analysis.*** Sense ***(B)*** and antisense ***(A)*** *SpPou4f2* probes were co-expressed together with SpOpsin4 antibody to analyze the sensitivity of *SpPou4f2* to probes. Both *SpPou4f2* and *SpPax6* antisense probes ***(A,C)*** generated unique expression patterns relative to SpOpsin 4 antibody whereas sense *SpPou4f2* probe did not yield any detectable signal ***(B)***.

Supplementary Materials and Methods

(a) RGC axon anterograde tracing

Two μl of Alexa-488 conjugated cholera toxin subunit (CTB488) (1 mg/ml, Invitrogen) was injected into the vitreous using a 35-gauge NanoFil system (World Precision Instruments) to trace the RGC axons in the brains. Three days after CTB injection, animals were anesthetized and sacrificed with perfusion using 4% PFA. Whole brains were dissected and post-fixed with 4% PFA. Cryo-preserved brains were sectioned into consecutive 100-μm coronal sections for imaging.

(b) Alkaline phosphatase (AP) staining

AP staining of brain sections was performed following procedures in Badea et al. with slight modification [2, 3]. In brief, brain sections were incubated in 65^oC to inactivate endogenous AP activity, and then incubated in AP staining solution [0.1 M Tris (pH9.5), 0.1 M NaCl, 50mM MgCl2, 3.4 μg/ml nitroblue tetrazolium (NBT), and 1.75 μg/ml 5-bromo-4-chloro-3-indolyl-phosphate (BCIP)]. Sections were then cleared in ScaleA2 reagent for 2 days [4M urea, 10% (vol/vol) glycerol, and 0.1% (vol/vol) Triton X-100] [4].

(c) ERG recordings

Animals were initially anesthetized with an intraperitoneal injection of ketamine (60 mg/kg) and xylazine (6 mg/kg), and anesthesia was maintained with subcutaneous injections of ketamine (30 mg/kg) and xylazine (3 mg/kg) every 30 to 40 minutes via a subcutaneous needle fixed in the flank. The animal's head was fixed in a metal head holder that also served as the electrical ground. Pupils were fully dilated to about 3 mm in diameter in all three genotypes with topical atropine (0.5%) and phenylephrine

(2.5%). Body temperature was monitored and maintained at 37°C with a thermostatically controlled electrically heated blanket (CWE, Inc.). Full-field flash ERGs were recorded using DTL fiber electrodes [5] placed across the center of the cornea of the tested eye and the fellow eye, which served as the reference for the low signal to noise responses to low stimulus strengths; a platinum pin inserted under the skin of the temple served as the reference when higher stimulus strengths were used. The cornea of the tested eye was covered with a contact lens heat-formed from clear film (Aclar). The non-tested eye was covered with a black plastic (PVC) contact lens and a black aluminum foil cap that covered both the eye and the skull to block any scattered light.

The range of stimulus strengths over which a- and b-waves were measured are included in Suppl. table 1. Positive (p) and negative (n) scotopic threshold response (STR) amplitudes were measured over the range of -6.5 to -4.4 log sc cd-s/m^2 where stimuli were too weak to elicit an a- or b-wave. pSTR amplitudes were measured from baseline to positive peak, and nSTR amplitudes were measured from baseline to trough of the slow negative wave that was maximal between 200 and 250 ms after the flash. Implicit times (ms) of the waveforms were measured as the time following flash onset to the peak for the b-wave and pSTR, and to the trough for the nSTR. Repeated-measures analysis of variance (SPSS, IBM) was performed to compare the stimulus response functions, using the Huynh-Feldt correction for non-sphericity. If a significant interaction (flash strength x genotype, $p \leq 0.05$) was detected across the three groups, a posthoc Tukey's test was done to determine which specific genotypes differed from each another.

1. Fu X., Kiyama T., Li R., Russell M., Klein W.H., Mu X. 2009 Epitope-tagging Math5 and Pou4f2: new tools to study retinal ganglion cell development in the mouse. *Developmental dynamics : an official publication of the American Association of Anatomists* **238**(9), 2309-2317. (doi:10.1002/dvdy.21974).
2. Badea T.C., Hua Z.L., Smallwood P.M., Williams J., Rotolo T., Ye X., Nathans J. 2009 New mouse lines for the analysis of neuronal morphology using CreER(T)/loxP-directed sparse labeling. *PloS one* **4**(11), e7859. (doi:10.1371/journal.pone.0007859).
3. Badea T.C., Nathans J. 2004 Quantitative analysis of neuronal morphologies in the mouse retina visualized by using a genetically directed reporter. *The Journal of comparative neurology* **480**(4), 331-351. (doi:10.1002/cne.20304).
4. Hama H., Kurokawa H., Kawano H., Ando R., Shimogori T., Noda H., Fukami K., Sakaue-Sawano A., Miyawaki A. 2011 Scale: a chemical approach for fluorescence imaging and reconstruction of transparent mouse brain. *Nature neuroscience* **14**(11), 1481-1488. (doi:10.1038/nn.2928).
5. Dawson W.W., Trick G.L., Litzkow C.A. 1979 Improved electrode for electroretinography. *Investigative ophthalmology & visual science* **18**(9), 988-991.
